# Role of C5b-9 and RGC-32 in Cancer

**DOI:** 10.3389/fimmu.2019.01054

**Published:** 2019-05-09

**Authors:** Sonia I. Vlaicu, Alexandru Tatomir, Violeta Rus, Horea Rus

**Affiliations:** ^1^Department of Internal Medicine, “Iuliu Hatieganu” University of Medicine and Pharmacy, Cluj-Napoca, Romania; ^2^Department of Neurology, School of Medicine, University of Maryland, Baltimore, MD, United States; ^3^Division of Rheumatology and Immunology, Department of Medicine, School of Medicine, University of Maryland, Baltimore, MD, United States

**Keywords:** C5b-9, cancer, RGC-32, cell proliferation, apoptosis

## Abstract

The complement system represents an effective arsenal of innate immunity as well as an interface between innate and adaptive immunity. Activation of the complement system culminates with the assembly of the C5b-9 terminal complement complex on cell membranes, inducing target cell lysis. Translation of this sequence of events into a malignant setting has traditionally afforded C5b-9 a strict antitumoral role, in synergy with antibody-dependent tumor cytolysis. However, in recent decades, a plethora of evidence has revised this view, highlighting the tumor-promoting properties of C5b-9. Sublytic C5b-9 induces cell cycle progression by activating signal transduction pathways (e.g., Gi protein/ phosphatidylinositol 3-kinase (PI3K)/Akt kinase and Ras/Raf1/ERK1) and modulating the activation of cancer-related transcription factors, while shielding malignant cells from apoptosis. C5b-9 also induces Response Gene to Complement (RGC)-32, a gene that contributes to cell cycle regulation by activating the Akt and CDC2 kinases. RGC-32 is expressed by tumor cells and plays a dual role in cancer, functioning as either a tumor promoter by endorsing malignancy initiation, progression, invasion, metastasis, and angiogenesis, or as a tumor suppressor. In this review, we present recent data describing the versatile, multifaceted roles of C5b-9 and its effector, RGC-32, in cancer.

## Introduction

Carcinogenesis in human somatic cells involves a series of genetic and epigenetic alterations that culminate in the generation of a malignant tissue fully prepared to elude most anticancer defense strategies. To date, there are seven key alterations in cancerous cells: self-sufficiency in growth signals; insensitivity to growth suppressors; evasion of apoptosis, enabling replicative immortality; sustained angiogenesis; tissue invasion (metastasis) (1) and the presence of cancer-related inflammation (CRI) (2). Essential orchestrators of CRI are the tumor-associated inflammatory cells (macrophages, fibroblasts, T cells and myeloid-derived suppressor cells) and their secreted chemokines and cytokines, along with complement activation within the tumor microenvironment (2, 3).

The complement system represents an effective arsenal of innate immunity as well as an interface between innate and adaptive immunity. An ancestral instrument in fighting invasive pathogens and efficient clearance of debris, the complement system can be activated by the classical, alternative, or lectin pathway, all of which unite at the level of C3 activation. All three pathways lead to the membrane attack complex formation and to cell lysis. Activation of the terminal complement proteins C5 to C9 generates membrane-inserted complexes C5b-7, C5b-8, and finally C5b-9, the so-called membrane attack complex (MAC) (4, 5).

Evidence supporting complement activation, in association with C5b-9 deposits during the antitumoral response exists for a variety of human malignancies (6). Niculescu et al. provided the first immunohistochemical support for the presence of C5b-9 deposits (along with IgG, C3 and C4 deposits) in a human cancerous tissue, namely breast carcinoma (3). Thereafter, numerous studies demonstrated C5b-9 deposition indicating complement activation in human thyroid (7), ovarian (8), endometrial (6), gastric (6–10), liver (6), colon, renal, and lung carcinomas (11), as well as in human osteosarcoma (12), medulloblastoma (6), glioma (6, 13), and gastrointestinal stromal tumor (6) tissues. High levels of soluble C5b-9 were also detected in the ascitic fluid of ovarian cancer (14). Elevated circulating C9 protein levels have been reported in the serum or plasma of colon (15) and gastric (16) adenocarcinoma, oral squamous cell carcinoma (17) and squamous cell lung cancer (18) patients.

C5b-9 has been shown to possess antitumoral properties, acting in synergy with monoclonal antibody (mAb)-based immunotherapies, many of which use complement activation and C5b-9 as an effector to kill tumor cells (19, 20). In this context, the mAb triggers C5b-9 assembly on cells leading to tumor destruction. Nevertheless, in recent decades, a plethora of evidence has brought about a conceptual switch in this paradigm (21) and exposed the tumor-promoter properties of C5b-9.

Here, we summarize the available data concerning the complex and versatile role of C5b-9, and that of its pivotal effector RGC-32, in cancer.

## Effects of Lytic C5b-9 on Tumor Cells

Successful achievement of cell lysis during complement-dependent cytotoxicity (CDC) requires the formation of multiple C5b-9 complexes on the cell surface (22). Once malignant Ehrlich ascites cells already bearing C5b-8 complexes are exposed to C9, a rapid and extensive ATP depletion, coupled with leakage of the adenine nucleotides ATP, ADP, and AMP, precedes cell death. Other prelytic events include the loss of mitochondrial membrane potential with consequent defective ATP synthesis and a vigorous Ca^2+^ influx, which initiate necrotic cell death (23).

The morphologic and biochemical changes induced by lytic MAC attack do share some features with those seen in apoptosis (nucleolar changes), although most features correspond more closely to necrotic changes (loss of volume control and defective mitochondria) (5, 24). The main biochemical changes include Bid cleavage, caspase activation, and activation of extracellular DNases (25–27). The impact of lytic C5b-9 on the malignant signaling pathways is multifaceted, since CDC has been documented to use several necrotic cell death pathways involving the receptor-interacting protein kinase 1 (RIPK1), receptor-interacting protein kinase 3 (RIPK3) and mixed-lineage kinase domain-like protein (MLKL) (28), in concert with the effectors JNK and Bid. The RIPK1/RIPK3/MLKL pathway closely resembles TNF-alpha-induced necroptosis (28).

Tumor cells have developed complement resistance through C5b-9 removal (29), expression of membrane complement-regulatory proteins (mCRPs) and other cell surface-protective molecules, and secretion of soluble complement inhibitors (30, 31). The ability of a cell to survive an initial complement-mediated membrane attack affords its resistance against future attacks (32).

The use of mAb-based immunotherapies that stimulate the destruction of tumor cells by CDC has received a lot of interest (19). The quest for optimal efficacy in CDC has incited many research teams. For instance, Diebolder et al. have shown that IgG hexamerization after antigen binding leads to more effective complement activation and fixation, and thus a more potent CDC (33). Narrow C5b-8 pores formed without C9 are sufficient for CDC due to efficient antibody-mediated hexamer formation (34). By neutralizing mCRP expression on leukemia cells, Mamidi et al. were the first to achieve both enhanced CDC and improved complement-dependent cellular cytotoxicity by monocyte-derived macrophages and macrophages induced by two anti-CD20 antibodies (rituximab and ofatumumab) and one anti-CD52 antibody (alemtuzumab) (35). Of late, the miR-200b, miR-200c, and miR-217 microRNAs have been recognized as potential regulators of mortalin as well as CD46 and CD55 expression in leukemia/ lymphoma and have been observed to coordinate the quantity of C5b-9 deposited on target cells (36).

## Sublytic C5b-9 Induces Tumor Cell Proliferation and Transcriptional Activation in Malignant Cells

**Figure 1:**
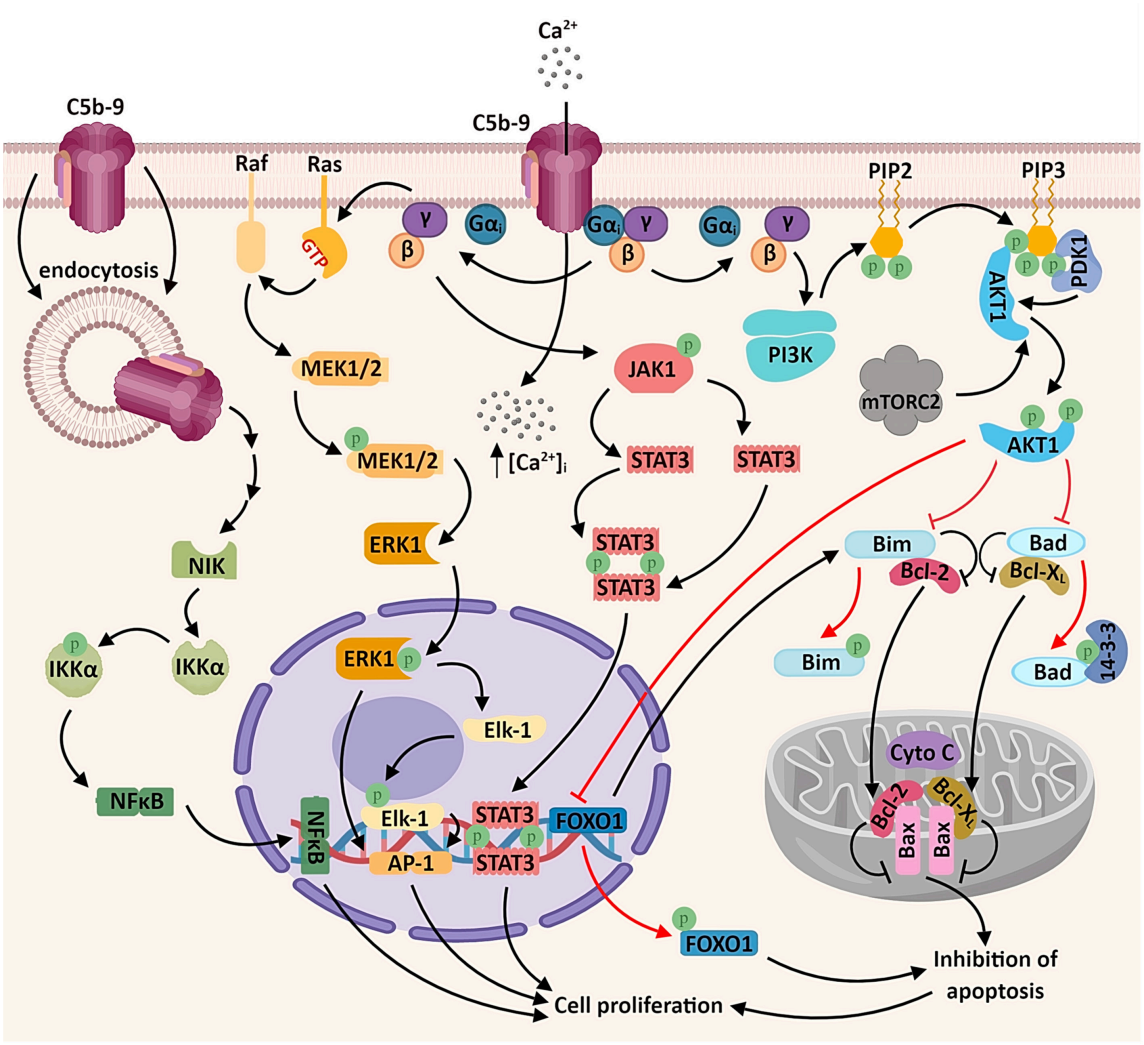
Sublytic C5b-9 promotes tumor growth and survival by activating several signaling pathways. The assembly of C5b-9 complexes in the cellular membrane activates the heterotrimeric G proteins of the G_i_ subtype (38). The β-γ complex is thought to activate several intracellular signaling cascades, including: a) phosphatidylinositol 3 kinase (PI3K)-Akt signaling pathway. Activated Akt phosphorylates and inactivates the pro-apoptotic factors Bad and Bim, resulting in the release of the pro-survival factors Bcl-2 and Bcl-xL, which migrate into the mitochondrial matrix and inhibit the release of cytochrome C (cyto C), thus inhibiting apoptosis. Akt also phosphorylates the transcription factor FOXO1, promoting its nuclear exclusion and inhibiting FOXO1-mediated transcription of pro-apoptotic factors (6, 39); b) Ras-Raf-MEK1-ERK1 signaling pathway, resulting in the activation of transcription factors associated with cell proliferation (AP-1 and Elk1); c) JAK1-STAT3 signaling pathway, leading to the formation of STAT3 homodimers and their nuclear translocation (6, 39–41). Another recently described mechanism which may account for the activation of genes associated with cell proliferation is the activation of the non-canonical NF-κB signaling pathway by the endosomal C5b-9 complexes, involving the NF-κB-inducing kinase (NIK) (42). An important consequence of C5b-9 assembly is the increased concentration of cytosolic calcium ions (Ca^2+^), either through direct entry of extracellular Ca^2+^ or by endoplasmic reticulum release triggered by intracellular second messengers (43) (not shown).

Sublytic levels of C5b-9 assembly in the membrane of malignant cells generate several different biological responses: activation of signal transduction pathways, proliferation, and modulation of apoptosis (5, 37) (Figure 1).

One of the first investigations of C5b-9 looked at the generation of signal messengers in Ehrlich carcinoma cells by the sublytic terminal complement complexes (TCC) C5b-9, C5b-8, and C5b-7 and identified the signal messengers involved in eliminating TCC from the cell surface (44). Exposure of Ehrlich carcinoma cells to C5b-9 caused an increase in cytosolic Ca^2+^. In addition, sublytic C5b-9 and C5b-8 substantially increased PKC activity, and C5b-8 and C5b-7 induced an increase in cAMP (44). In another report, sublytic C5b-9 assembly in lymphoblastoid human B cells stimulated the production of diacylglycerol (DAG) and ceramide (mediators of inflammation and tissue repair), along with PKC activation (45). Rapid elimination of TCC from the membrane surface was inhibited by pretreatment with pertussis toxin, suggesting the involvement of a G_i_ protein (38).

One of the pioneer studies on sublytic C5b-9-induced tumor cell proliferation through mitotic signaling (40) demonstrated that a significant increase in DNA synthesis over the C5b6 level is induced by C5b-9 in the human lymphoblastoid B cell line JY 25. This effect (but not the basal DNA synthesis activity) could be abrogated by pertussis toxin pretreatment, indicating the involvement of activated G_i_ proteins in DNA synthesis induced by C5b-9 in tumor cells (40) (Figure 1). Pretreatment of cells with PD98059 (specific inhibitor of MEK1 activation) was also effective in abolishing C5b-9-induced DNA synthesis (40). Both ERK1, a member of a potentially pro-oncogenic signal transduction pathway, and PI3K contribute to the transmission of downstream cellular effects prompted by sublytic C5b-9 (40, 46, 47). Indeed, as shown by Pilzer et al., C5b-9 deposition on the K562 leukemic cell membrane activates PKC and ERK protein kinases (48), which then induce the relocation of the mitochondrial chaperone mortalin from the mitochondria to the plasma membrane, where mortalin escorts exo-vesiculated C5b-9 complexes (49). Co-localization of mortalin and C5b-9 in distinct puncta at the leukemic cell plasma membrane region has also been well-documented (49). Mortalin is overexpressed in a multitude of malignancies, and a high level of circulating mortalin was recently demonstrated to correlate with high mortality in colorectal cancer patients (50). Mortalin supports the process of carcinogenesis by suppressing pathway-mediated growth-inhibitory signaling, inactivating tumor suppressor p53, and activating epithelial-to-mesenchymal transition (EMT) signaling (51). In addition, Rozenberg et al. has found that HSP90 binds to mortalin and protects cells from complement-mediated cytotoxicity by inhibiting, together with mortalin, C5b-9 assembly on the plasma membrane (52).

Among the signal transduction networks regulating cancer progression that have been found to function downstream of sublytic C5b-9 are p38/MAPK/JNK1 and JAK1/STAT3 (39, 41). Cellular proliferation induced by membrane-inserted sublytic C5b-9 relies on the activation of the Gi protein/PI3K/Akt kinase and Ras/Raf-1/ERK1 pathways and regulation of cell cycle-specific genes and proto-oncogenes (5, 47) (Figure 1).

Of note, activation of activating protein 1 (AP-1) transcription factor has also been documented following C5b-9 treatment of lymphoblastoid B-cell lines (40). Consistent with this finding, stimulation with C5b-9 enhances the expression of the oncogenic proteins c-jun, JunD, and c-fos (53). AP-1 functions are dependent on the specific Fos and Jun subunits contributing to AP-1 dimers (54). AP-1 activity is crucial to oncogenesis, and there is evidence that it has an ambivalent role: while it can act as a tumor promoter in some cancer types, it also represses tumor formation in others (54). NF-κB is another major transcription factor known to be activated by sublytic C5b-9 (42, 47, 55). Sublytic C5b-9–induced, NF-κB–regulated proteins may further enhance cell survival (56) (Figure 1).

In smooth muscle cells (SMC), sublytic assault by MAC stimulates release of insulin-like growth factor-1 (57), whereas in glomerular epithelial cells it causes transactivation of the receptors for epidermal growth factor (EGF), human epidermal growth factor 2/Neu, fibroblast growth factor, and hepatocyte growth factor, all vital growth factors during tumor development (58). CT26 colon carcinoma cells exposed to sublytic C5b-9 exhibit significant changes in genes involved in Ca^2+^ and G-protein signal transduction, early response transcription factors (EGR1, EGR2) and four genes encoding proteins with extracellular localization: AREG, CXCL1, MMP3, and MMP13 (59). Network analysis has suggested an important role for the EGF receptor as the main canonical signaling cascade in the response to sublytic C5b-9 in the colon carcinoma cells (59). This connection is very pertinent to carcinogenesis, since alterations in EGF receptor signaling are common events in several human cancers (60).

Non-lethal C5b-9 activates cell cycle by directly influencing major cell cycle regulators: in aortic SMC, sublytic C5b-9 increases the activity of the cyclin-dependent kinases CDK4 and CDK2, whereas in endothelial cells it increases the levels of cell division cycle protein 2 (CDC2), cyclin D1, and proliferating cell nuclear antigen (PCNA) (39).

## Sublytic C5b-9 Protects Tumor Cells From Apoptosis

Sublytic complement-induced protection against TNF-α-mediated apoptosis accompanies the induction of the anti-apoptotic proteins Bcl-2 and Bcl-xL, along with suppressing the TNF-α-induced decrease in the amount of Bcl-2 and Bcl-xL (61, 62). The anti-apoptotic effects of sublytic C5b-9 encompass events such as activation of NF-kB and inhibition of caspase-8 activation (61, 63, 64). Fascinating insight into the relationship of microvesicles to apoptosis has been provided by the work of Stratton et al. in prostate cancer cells (43). Sublytic C5b-9 deposition is among the positive signals that result in a high Ca^2+^ cellular influx and membrane depolarization; stimulation of microvesicle release then ensures shedding of excess intracellular calcium and export of damaging agents such as deposited C5b-9 and caspase-3. This circuit provides cells with an effective mechanism to thwart apoptosis (43).

It should be noted, however, that data also exist in support of the ability of sublytic C5b-9 to activate various molecules that potentially contribute to programed cellular death. In lung epithelial cells, MAC insertion has been observed to induce Ca^2+^ influx, leading to mitochondrial overload and loss of mitochondrial transmembrane potential. These changes prompt NLPR3 inflammasome activation, as well as IL-1β production, cytoplasmatic cytochrome c release and caspase activation (65). A similar chain of events has been described in macrophages in which “bystander” deposition of MAC on the plasma membranes of phagocytic macrophages incite NLRP3 inflammasome and caspase-1 activation, together with IL-1β and IL-18 release (66). Bystander C5b-9 deposition has also been found to modulate T-cell polarization and leucocyte recruitment to the phagocytic sites (66). Despite the analogy with apoptosis, the involvement of NLRP3 inflammasomes, caspase-1, IL-1β, and IL-18 rather evokes another form of programmed cellular death, pyroptosis (67). While activation of pyroptosis provides powerful ammunition against many types of cancers, other researchers have reported that the NLRP3 inflammasome and IL-1β pathway promote cancer progression in animal and human breast cancer models (68) and asbestos-induced malignant mesothelioma (69). In addition, sublytic C5b-9 has been shown to interact with effectors of TNFα-induced necroptosis, yet another type of programmed cell death: exposure of human erythroleukemia K562 cells to sublytic C5b-9 causes the activation of RIPK1, RIPK3, and MLKL, co-localization of RIPK3 with RIPK1 in the cytoplasm and co-localization of RIPK3 and MLKL with C5b-9 at the plasma membrane (28). The meaning of the association between C5b-9 and necroptotic effectors has many nuances: RIPK3 and MLKL are in fact seen as putative tumor suppressors (70), but *in vitro* work in breast cancer cells has recently highlighted the contributions of the necroptotic genes RIPK1, RIPK3, and MLKL in promoting anchorage-independent tumor growth and mediating tumor cell resistance to radiation (71).

## C5b-9 and Angiogenesis

Although initiated by cellular destruction and hypoxia, the propagation of the vascular network in a malignant environment is sustained by upregulation of pro-angiogenic factors (e.g. vascular endothelial growth factor [VEGF], TGF-α, TGF-β, TNF-α, EGF, fibroblast growth factor [FGF]) and downregulation of negative angiogenic regulators (IL-10, IL-12, angiopoietin-2, angiotensin) (72).

Accelerated C5b-9 deposition, accompanied by VEGF, β-FGF, and TGF-β2 release is seen during laser-induced choroidal neovascularization in age-related macular degeneration in CD59-deficient mice (73). Likewise, exposure of retinal pigment epithelium cells to oxidative stress has been found to induce sublytic C5b-9 activation, triggering VEGF secretion via the Src and Ras-Erk pathways (74).

The effects of C5b-9 were later corroborated in cancer cells. In an osteosarcoma epithelial cell line, sublytic C5b-9 activation (via the alternative pathway) instigated production of angiogenic growth factors FGF1 and VEGF-A via the ERK signaling pathway (12).

## RGC-32 and Cancer

The RGC-32 gene was first cloned from rat oligodendrocytes via differential display by Badea and coworkers, in their quest to identify the genes differentially expressed in response to sublytic complement activation (75, 76). RGC-32 fundamentally regulates cellular processes such as the cell cycle, differentiation, wound healing and tumorigenesis (75, 77). It directly binds to cyclin-dependent kinase CDC2 and Akt and stimulates their kinase activity (75, 78).

Various studies have described an aberrant RGC-32 mRNA expression in human cancers: up-regulation in colon (79, 80), ovarian (81, 82), breast (79, 83, 84) and prostate (79) cancers and lymphomas (85, 86) and downregulation in glioblastomas (87), astrocytomas (88), adrenocortical carcinomas (89), and multiple myelomas (90).

We have originally demonstrated a role for RGC-32 deregulation in colon adenocarcinoma, showing that the intensity of RGC-32 immunohistochemical staining corresponded to the increase in the TNM staging of the adenocarcinomas (77). Later, the expression of RGC-32 was shown to be up-regulated in pancreatic cancer tissues and to correlate with TNM stages (91).

**Figure 2:**
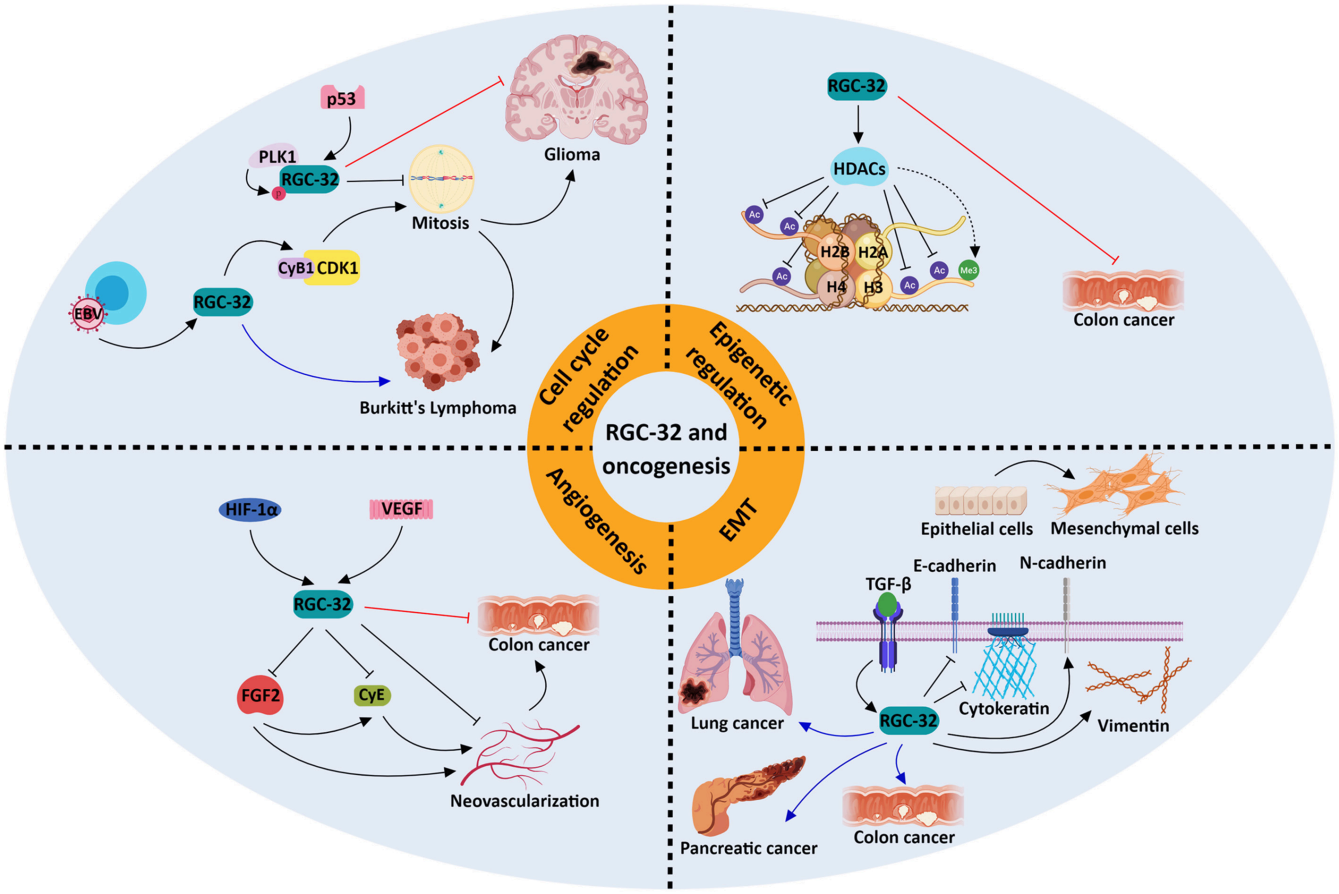
Molecular mechanisms underlying the role of RGC-32 in oncogenesis. RGC-32 can act both as a tumor suppressor (red inhibitory lines) and a tumor promoter (blue arrows) in a variety of cancers by activating a plethora of molecular pathways. RGC-32 plays an important role in: (a) promoting the TGF-β-induced epithelial-to-mesenchymal transition (EMT), a process in which epithelial cells lose their adhesiveness and gain myofibroblast-like phenotypes, inducing metastasis and cancer progression (80, 91, 92); (b) epigenetic modifications, by inducing histone deacetylases (HDACs), which in turn deacetylate various histone targets such as H2B at lysine 5 (H2BK5), H2BK15, H3K9, and H4K8 and indirectly promote the tri-methylation of H3K27. This in turn may result in transcriptional repression of genes associated with cancer progression (77); (c) cell cycle regulation, in which RGC-32 can promote mitosis by enhancing the activity of kinases crucial for cell cycle progression (93), or induce cell cycle arrest in a p53-dependent manner (88); (d) inhibition of angiogenesis, in which it may behave as a negative feedback regulator of hypoxia-induced signaling pathways (94). The involvement of RGC-32 in these processes might explain its apparent dual role as a tumor suppressor/promoter in the same type of tumor, such as colon cancer.

Using a gene array and SW480 colon adenocarcinoma cells, we have identified groups of genes that are significantly changed by RGC-32 silencing (77), including genes implicated in chromatin assembly, cell cycle, and RNA processing. We have observed increased lysine acetylation at multiple sites on histones H2B, H3, and H4, and lessened expression of the histone deacetylase SIRT1 upon silencing of RGC-32 expression in SW480 cells (77) (Figure 2). Moreover, an absence of RGC-32 expression induces DNA synthesis and mitosis in colon cancer cells (77). Correspondingly, overexpression of RGC-32 in several cancer cell lines has been shown to delay G2/M cell cycle progression (88).

On the other hand, others have reported that RGC-32 promotes malignant cell proliferation in the colon adenocarcinoma cell line SW480 (80) and in lung adenocarcinoma LTE cells (92). Overexpression of RGC-32 protein in Epstein Barr virus (EBV)-immortalized B cells has been found to disrupt the G2/M checkpoint via CDK1 activation, and RGC-32 has been shown to be indispensable for the growth and survival of lymphoblastoid B cells (86, 93) (Figure 2).

The cooperation of RGC-32 with SMAD3, as TGF-β downstream effectors, in the regulation of EMT seen in renal tubular cells (95) indicates a possible involvement of RGC-32 in invasion and metastasis. RGC-32 was shown to influence expression of vimentin, cadherin, and the transcription factors Snail and Slug in pancreatic and colon cancer lines (80, 91) (Figure 2). Also, excessive RGC-32 expression in a colon cancer cell line prompts cytoskeleton reorganization and cell migration (96). Similarly, RGC-32 has been demonstrated to induce EMT and to promote cancer cell migration and invasion in lung adenocarcinoma cells via decreases in the protein level and activity of the matrix metalloproteinases MMP-2 and MMP-9 (92, 97).

Research focusing on the effects of RGC-32 in animal cancer models has yielded contrasting data. Colon cancer tumors lacking RGC-32 that were implanted into nude mice were observed to have a lower growth rate and significantly smaller tumor volumes than did the tumors with intact RGC-32 expression (80). In striking contrast, inoculating RGC-32 into colon cancer tumors placed subcutaneously in mice resulted in a significant tumor growth suppression and decline in angiogenesis (94) (Figure 2).

The thesis of RGC-32 as a functional dyad (tumor suppressor/ promoter) accounts for its contradictory behavior during cancerogenesis: the protein acts in a pleiotropic manner in distinct malignant settings, dependent on the cellular lineage and on the various ligands. For instance, RGC-32 exerts a tumor-suppressive effect in lung adenocarcinomas with wild-type *TP53*, but a tumor-promoting effect in the tumors carrying *TP53* mutations (98). Targeting RGC-32 should be done in conjunction with the role played in specific tumors as well as by using biomarkers that can predict the efficacy of RGC-32 inhibitors in cancer patients. Future studies are needed in order to find effective RGC-32-based drugs.

## Conclusions

Considering all the available data, the role of C5b-9 in cancer is indisputably versatile: while it is lethal to tumor cells in a lytic context, when C5b-9 becomes activated to a sublytic level, it instead stimulates tumor growth through several mechanisms. Counteracting these tumor-promoting traits of C5b-9 by therapeutically surmounting CDC resistance in cancer cells and potentiating the antitumoral actions of C5b-9 (and therefore the efficacy of mAb-based immunotherapy) constitutes the next major direction in the field of immuno-oncology.

## Author Contributions

HR designed the study. SV, AT, VR, and HR wrote the manuscript. All authors approved the manuscript.

### Conflict of Interest Statement

The authors declare that the research was conducted in the absence of any commercial or financial relationships that could be construed as a potential conflict of interest.

## Abbreviations

AP-1: activator protein 1
CDC: complement-dependent cytotoxicity
CDK1: cyclin–dependent kinase 1
CyB1: cyclin B1
CyE: cyclin E
EBV: Epstein-Barr virus
EGF: epidermal growth factor
ERK1: extracellular signal-regulated kinase 1
FGF2: fibroblast growth factor 2
HIF-1α: hypoxia inducible factor 1 alpha
IKKα: inhibitor of nuclear factor kappa-B kinase subunit alpha
JAK1: Janus kinase 1
MAC: membrane attack complex
MEK1/2: mitogen activated protein kinase kinase 1
mTORC2: mammalian target of rapamycin complex 2
NF-κB: nuclear factor kappa-light-chain-enhancer of activated B cells
PLK1: polo-like kinase 1
RGC-32: response gene to complement 32
RIPK: receptor-interacting protein kinase
TCC: terminal complement complex
VEGF: vascular endothelial growth factor
STAT3: signal transducer and activator of transcription 3.

## References

[1] Hanahan D Weinberg RA. Hallmarks of cancer: the next generation. Cell. (2011) 144:646–74. 10.1016/j.cell.2011.02.013 21376230

[2] Balkwill FR Mantovani A. Cancer-related inflammation: common themes and therapeutic opportunities. Semin Cancer Biol. (2012) 22:33–40. 10.1016/j.semcancer.2011.12.005 22210179

[3] Niculescu F Rus HG Retegan M Vlaicu R. Persistent complement activation on tumor cells in breast cancer. Am J Pathol. (1992)140:1039–43. 1374587 PMC1886512

[4] Shin ML Rus HG Niculescu FI Membrane attack by complement: assembly and biology of terminal complement complexes. In: Lee AG, editor. Biomembranes: A Multi-Volume Treatise. Volume 4. JAI. Greenwich, CT: JAI Press (1996). p. 123–49.

[5] Tegla CA Cudrici C Patel S Trippe R III Rus V Niculescu F. Membrane attack by complement: the assembly and biology of terminal complement complexes. Immunol Res. (2011) 51:45–60. 10.1007/s12026-011-8239-5 21850539 PMC3732183

[6] Vlaicu SI Tegla CA Cudrici CD Danoff J Madani H Sugarman A. Role of C5b-9 complement complex and response gene to complement-32 (RGC-32) in cancer. Immunol Res. (2013) 56:109–21. 10.1007/s12026-012-8381-8 23247987

[7] Yamakawa M Yamada K Tsuge T Ohrui H Ogata T Dobashi M. Protection of thyroid cancer cells by complement-regulatory factors. Cancer. (1994) 73:2808–17. 7514955 10.1002/1097-0142(19940601)73:11<2808::aid-cncr2820731125>3.0.co;2-p

[8] Cho MS Vasquez HG Rupaimoole R Pradeep S Wu S Zand B. Autocrine effects of tumor-derived complement. Cell Rep. (2014) 6:1085–95. 10.1016/j.celrep.2014.02.014 24613353 PMC4084868

[9] Chen J Yang WJ Sun HJ Yang X Wu YZ. C5b-9 Staining Correlates With Clinical and Tumor Stage in Gastric Adenocarcinoma. Appl Immunohistochem Mol Morphol. (2016) 24:470–5. 10.1097/PAI.0000000000000218 26186252 PMC4979623

[10] Inoue T Yamakawa M Takahashi T. Expression of complement regulating factors in gastric cancer cells. Mol Pathol. (2002) 55:193–9. 10.1136/mp.55.3.193 12032231 PMC1187173

[11] Niehans GA Cherwitz DL Staley NA Knapp DJ Dalmasso AP. Human carcinomas variably express the complement inhibitory proteins CD46 (membrane cofactor protein), CD55 (decay-accelerating factor), and CD59 (protectin). Am J Pathol. (1996) 149:129–42. 8686736 PMC1865231

[12] Jeon H Han SR Lee S Park SJ Kim JH Yoo SM. Activation of the complement system in an osteosarcoma cell line promotes angiogenesis through enhanced production of growth factors. Sci Rep. (2018) 8:5415. 10.1038/s41598-018-23851-z 29615744 PMC5883033

[13] Bouwens TA Trouw LA Veerhuis R Dirven CM Lamfers ML Al-Khawaja H. Complement activation in Glioblastoma multiforme pathophysiology: evidence from serum levels and presence of complement activation products in tumor tissue. J Neuroimmunol. (2015) 278:271–6. 10.1016/j.jneuroim.2014.11.016 25468776

[14] Bjorge L Hakulinen J Vintermyr OK Jarva H Jensen TS Iversen OE. Ascitic complement system in ovarian cancer. Br J Cancer. (2005) 92:895–905. 10.1038/sj.bjc.6602334 15726105 PMC2361909

[15] Murakoshi Y Honda K Sasazuki S Ono M Negishi A Matsubara J. Plasma biomarker discovery and validation for colorectal cancer by quantitative shotgun mass spectrometry and protein microarray. Cancer Sci. (2011) 102:630–8. 10.1111/j.1349-7006.2010.01818.x 21199170

[16] Chong PK Lee H Loh MC Choong LY Lin Q So JB. Upregulation of plasma C9 protein in gastric cancer patients. Proteomics. (2010) 10:3210–21. 10.1002/pmic.201000127 20707004 PMC3760195

[17] Gallenkamp J Spanier G Worle E Englbrecht M Kirschfink M Greslechner R. A novel multiplex detection array revealed systemic complement activation in oral squamous cell carcinoma. Oncotarget. (2018) 9:3001–13. 10.18632/oncotarget.22963 29423024 PMC5790441

[18] Narayanasamy A Ahn JM Sung HJ Kong DH Ha KS Lee SY. Fucosylated glycoproteomic approach to identify a complement component 9 associated with squamous cell lung cancer (SQLC). J Proteomics. (2011) 74:2948–58. 10.1016/j.jprot.2011.07.019 21840429

[19] Macor P Capolla S Tedesco F. Complement as a biological tool to control tumor growth. Front Immunol. (2018) 9:2203. 10.3389/fimmu.2018.02203 30319647 PMC6167450

[20] Taylor RP Lindorfer MA. The role of complement in mAb-based therapies of cancer. Methods. (2014) 65:18–27. 10.1016/j.ymeth.2013.07.027 23886909

[21] Reis ES Mastellos DC Ricklin D Mantovani A Lambris JD. Complement in cancer: untangling an intricate relationship. Nat Rev Immunol. (2018) 18:5–18. 10.1038/nri.2017.97 28920587 PMC5816344

[22] Koski CL Ramm LE Hammer CH Mayer MM Shin ML. Cytolysis of nucleated cells by complement: cell death displays multi-hit characteristics. Proc Natl Acad Sci USA. (1983). 80:3816–20. 6602341 10.1073/pnas.80.12.3816 PMC394143

[23] Papadimitriou JC Ramm LE Drachenberg CB Trump BF Shin ML. Quantitative analysis of adenine nucleotides during the prelytic phase of cell death mediated by C5b-9. J Immunol. (1991) 147:212–7. 1904901

[24] Papadimitriou JC Drachenberg CB Shin ML Trump BF. Ultrastructural studies of complement mediated cell death: a biological reaction model to plasma membrane injury. Virchows Arch. (1994) 424:677–85. 8055163 10.1007/BF00195784

[25] Cragg MS Howatt WJ Bloodworth L Anderson VA Morgan BP Glennie MJ. Complement mediated cell death is associated with DNA fragmentation. Cell Death Differ. (2000) 7:48–58. 10.1038/sj.cdd.4400627 10713720

[26] Gancz D Fishelson Z. Cancer resistance to complement-dependent cytotoxicity (CDC): Problem-oriented research and development. Mol Immunol. (2009) 46:2794–800. 10.1016/j.molimm.2009.05.009 19501402

[27] Ziporen L Donin N Shmushkovich T Gross A Fishelson Z. Programmed necrotic cell death induced by complement involves a Bid-dependent pathway. J Immunol. (2009) 182:515–21. 10.4049/jimmunol.182.1.515 19109183

[28] Lusthaus M Mazkereth N Donin N Fishelson Z. Receptor-interacting protein kinases 1 and 3, and mixed lineage kinase domain-like protein are activated by sublytic complement and participate in complement-dependent cytotoxicity. Front Immunol. (2018) 9:306. 10.3389/fimmu.2018.00306 29527209 PMC5829068

[29] Carney DF Koski CL Shin ML Elimination of terminal complement intermediates from the plasma membrane of nucleated cells: the rate of disappearance differs for cells carrying C5b-7 or C5b-8 or a mixture of C5b-8 with a limited number of C5b-9. J Immunol. (1985)134:1804–9. 3968432

[30] Bohana-Kashtan O Ziporen L Donin N Kraus S Fishelson Z. Cell signals transduced by complement. Mol Immunol. (2004) 41:583–97. 10.1016/j.molimm.2004.04.007 15219997

[31] Donin N Jurianz K Ziporen L Schultz S Kirschfink M Fishelson Z. Complement resistance of human carcinoma cells depends on membrane regulatory proteins, protein kinases and sialic acid. J Clin Exp Immunol. (2003) 131:254–63. 10.1046/j.1365-2249.2003.02066.x 12562385 PMC1808622

[32] Fishelson Z Donin N Zell S Schultz S Kirschfink M. Obstacles to cancer immunotherapy: expression of membrane complement regulatory proteins (mCRPs) in tumors. Mol immunol. (2003) 40:109–23. 10.1016/S0161-5890(03)00112-3 12914817

[33] Diebolder CA Beurskens FJ de Jong RN Koning RI Strumane K Lindorfer MA. Complement is activated by IgG hexamers assembled at the cell surface. Science. (2014) 343:1260–3. 10.1126/science.1248943 24626930 PMC4250092

[34] Taylor RP Lindorfer MA Cook EM Beurskens FJ Schuurman J Parren P. Hexamerization-enhanced CD20 antibody mediates complement-dependent cytotoxicity in serum genetically deficient in C9. Clin Immunol. (2017) 181:24–8. 10.1016/j.clim.2017.05.016 28578024

[35] Mamidi S Hone S Teufel C Sellner L Zenz T Kirschfink M. Neutralization of membrane complement regulators improves complement-dependent effector functions of therapeutic anticancer antibodies targeting leukemic cells. Oncoimmunology. (2015) 4:e979688. 10.4161/2162402X.2014.979688 25949896 PMC4404820

[36] Hillman Y Mazkereth N Farberov L Shomron N Fishelson Z. Regulation of complement-dependent cytotoxicity by microRNAs miR-200b, miR-200c, and miR-217. J Immunol. (2016) 196:5156–65. 10.4049/jimmunol.1502701 27183614

[37] Rus HG Niculescu FI Shin ML. Role of the C5b-9 complement complex in cell cycle and apoptosis. Immunol Rev. (2001) 180:49–55. 10.1034/j.1600-065X.2001.1800104.x 11414362

[38] Niculescu F Rus H Shin ML. Receptor-independent activation of guanine nucleotide-binding regulatory proteins by terminal complement complexes. J Biol Chem. (1994) 269:4417–23. 8308012

[39] Fosbrink M Niculescu F Rus H. The role of c5b-9 terminal complement complex in activation of the cell cycle and transcription. Immunol Res. (2005) 31:37–46. 10.1385/IR:31:1:37 15591621

[40] Niculescu F Rus H van Biesen T Shin ML. Activation of Ras and mitogen-activated protein kinase pathway by terminal complement complexes is G protein dependent. J Immunol. (1997) 158:4405–12. 9127005

[41] Niculescu F Soane L Badea T Shin M Rus H. Tyrosine phosphorylation and activation of Janus kinase 1 and STAT3 by sublytic C5b-9 complement complex in aortic endothelial cells. Immunopharmacology. (1999) 42:187–93. 10.1016/S0162-3109(99)00014-4 10408379

[42] Jane-wit D Surovtseva YV Qin L Li G Liu R Clark P. Complement membrane attack complexes activate noncanonical NF-kappaB by forming an Akt+ NIK+ signalosome on Rab5+ endosomes. Proc Natl Acad Sci USA. (2015) 112:9686–91. 10.1073/pnas.1503535112 26195760 PMC4534258

[43] Stratton D Moore C Antwi-Baffour S Lange S Inal J. Microvesicles released constitutively from prostate cancer cells differ biochemically and functionally to stimulated microvesicles released through sublytic C5b-9. Biochem Biophys Res Commun. (2015) 460:589–95. 10.1016/j.bbrc.2015.03.074 25817790

[44] Carney DF Lang TJ Shin ML. Multiple signal messengers generated by terminal complement complexes and their role in terminal complement complex elimination. J Immunol. (1990) 145:623–9. 2164064

[45] Niculescu F Rus H Shin S Lang T Shin ML. Generation of diacylglycerol and ceramide during homologous complement activation. J Immunol. (1993) 150:214–24. 8417124

[46] Niculescu F Badea T Rus H. Sublytic C5b-9 induces proliferation of human aortic smooth muscle cells: role of mitogen activated protein kinase and phosphatidylinositol 3-kinase. Atherosclerosis. (1999) 142:47–56. 10.1016/S0021-9150(98)00185-3 9920505

[47] Niculescu F Rus H. Mechanisms of signal transduction activated by sublytic assembly of terminal complement complexes on nucleated cells. Immunol Res. (2001) 24:191–9. 10.1385/IR:24:2:191 11594456

[48] Pilzer D Fishelson Z. Mortalin/GRP75 promotes release of membrane vesicles from immune attacked cells and protection from complement-mediated lysis. Int Immunol. (2005) 17:1239–48. 10.1093/intimm/dxh300 16091382

[49] Mazkereth N Rocca F Schubert JR Geisler C Hillman Y Egner A. Complement triggers relocation of Mortalin/GRP75 from mitochondria to the plasma membrane. Immunobiology. (2016) 221:1395–406. 10.1016/j.imbio.2016.07.005 27475989

[50] Jubran R Kocsis J Garam N Malati E Gombos T Barabas L. Circulating mitochondrial stress 70 protein/mortalin and cytosolic Hsp70 in blood: risk indicators in colorectal cancer. Int J Cancer. (2017) 141:2329–35. 10.1002/ijc.30918 28791678

[51] Yun CO Bhargava P Na Y Lee JS Ryu J Kaul SC. Relevance of mortalin to cancer cell stemness and cancer therapy. Sci Rep. (2017) 7:42016. 10.1038/srep42016 28165047 PMC5292728

[52] Rozenberg P Ziporen L Gancz D Saar-Ray M Fishelson Z. Cooperation between Hsp90 and mortalin/GRP75 in resistance to cell death induced by complement C5b-9. Cell Death Dis. (2018) 9:150. 10.1038/s41419-017-0240-z 29396434 PMC5833442

[53] Rus HG Niculescu F Shin ML. Sublytic complement attack induces cell cycle in oligodendrocytes. J Immunol. (1996) 156:4892–900. 8648139

[54] Gazon H Barbeau B Mesnard JM Peloponese JM Jr. Hijacking of the AP-1 Signaling pathway during development of ATL. Front Microbiol. (2017) 8:2686. 10.3389/fmicb.2017.02686 29379481 PMC5775265

[55] Viedt C Hansch GM Brandes RP Kubler W Kreuzer J. The terminal complement complex C5b-9 stimulates interleukin-6 production in human smooth muscle cells through activation of transcription factors NF-kappa B and AP-1. Faseb J. (2000) 14:2370–2. 10.1096/fj.00-0468fje 11024008

[56] Gancz D Lusthaus M Fishelson Z. A role for the NF-kappaB pathway in cell protection from complement-dependent cytotoxicity. J Immunol. (2012) 189:860–6. 10.4049/jimmunol.1103451 22685314

[57] Zwaka TP Torzewski J Hoeflich A Dejosez M Kaiser S Hombach V. The terminal complement complex inhibits apoptosis in vascular smooth muscle cells by activating an autocrine IGF-1 loop. Faseb J. (2003) 17:1346–8. 10.1096/fj.02-0814fje 12759337

[58] Cybulsky AV Takano T Papillon J McTavish AJ. Complement C5b-9 induces receptor tyrosine kinase transactivation in glomerular epithelial cells. Am J Pathol. (1999) 155:1701–11. 10.1016/S0002-9440(10)65485-5 10550326 PMC1866958

[59] Towner LD Wheat RA Hughes TR Morgan BP. Complement membrane attack and tumorigenesis: a systems biology approach. J Biol Chem. (2016) 291:14927–38. 10.1074/jbc.M115.708446 27226542 PMC4946912

[60] Sigismund S Avanzato D Lanzetti L. Emerging functions of the EGFR in cancer. Mol Oncol. (2018) 12:3–20. 10.1002/1878-0261.12155 29124875 PMC5748484

[61] Liu L Li W Li Z Kirschfink M. Sublytic complement protects prostate cancer cells from tumour necrosis factor-alpha-induced cell death. Clin Exp Immunol. (2012) 169:100–8. 10.1111/j.1365-2249.2012.04596.x 22774984 PMC3406369

[62] Soane L Rus H Niculescu F Shin ML. Inhibition of oligodendrocyte apoptosis by sublytic C5b-9 is associated with enhanced synthesis of bcl-2 and mediated by inhibition of caspase-3 activation. J Immunol. (1999) 163:6132–8. 10570303

[63] Cudrici C Niculescu F Jensen T Zafranskaia E Fosbrink M Rus V. C5b-9 terminal complex protects oligodendrocytes from apoptotic cell death by inhibiting caspase-8 processing and up-regulating FLIP. J Immunol. (2006) 176:3173–80. 10.4049/jimmunol.176.5.3173 16493077

[64] Soane L Cho HJ Niculescu F Rus H Shin ML. C5b-9 terminal complement complex protects oligodendrocytes from death by regulating Bad through phosphatidylinositol 3-kinase/Akt pathway. J Immunol. (2001) 167:2305–11. 10.4049/jimmunol.167.4.2305 11490019

[65] Triantafilou K Hughes TR Triantafilou M Morgan BP. The complement membrane attack complex triggers intracellular Ca2+ fluxes leading to NLRP3 inflammasome activation. J Cell Sci. (2013) 126:2903–13. 10.1242/jcs.124388 23613465

[66] Suresh R Chandrasekaran P Sutterwala FS Mosser DM. Complement-mediated ‘bystander' damage initiates host NLRP3 inflammasome activation. J Cell Sci. (2016) 129:1928–39. 10.1242/jcs.179291 27006116 PMC4893798

[67] Kolb JP Oguin TH III Oberst A Martinez J. Programmed cell death and inflammation: winter is coming. Trends Immunol. (2017) 38:705–18. 10.1016/j.it.2017.06.009 28734635 PMC5710799

[68] Guo B Fu S Zhang J Liu B Li Z. Targeting inflammasome/IL-1 pathways for cancer immunotherapy. Sci Rep. (2016) 6:36107. 10.1038/srep36107 27786298 PMC5082376

[69] Kadariya Y Menges CW Talarchek J Cai KQ Klein-Szanto AJ Pietrofesa RA. Inflammation-Related IL1beta/IL1R Signaling Promotes the Development of Asbestos-Induced Malignant Mesothelioma. Cancer Prev Res. (2016) 9:406–14. 10.1158/1940-6207.CAPR-15-0347 26935421 PMC4854753

[70] Krysko O Aaes TL Kagan VE D'Herde K Bachert C Leybaert L. Necroptotic cell death in anti-cancer therapy. Immunol Rev. (2017) 280:207–19. 10.1111/imr.12583 29027225

[71] Liu X Zhou M Mei L Ruan J Hu Q Peng J. Key roles of necroptotic factors in promoting tumor growth. Oncotarget. (2016) 7:22219–33. 10.18632/oncotarget.7924 26959742 PMC5008357

[72] Prager GW Poettler M. Angiogenesis in cancer. Basic mechanisms and therapeutic advances. Hamostaseologie. (2012) 32:105–14. 10.5482/ha-1163 21837355

[73] Bora NS Kaliappan S Jha P Xu Q Sivasankar B Harris CL. CD59, a complement regulatory protein, controls choroidal neovascularization in a mouse model of wet-type age-related macular degeneration. J Immunol. (2007) 178:1783–90. 10.4049/jimmunol.178.3.1783 17237428

[74] Kunchithapautham K Rohrer B. Sublytic Membrane-Attack-Complex (MAC) activation alters regulated rather than constitutive vascular endothelial growth factor (VEGF) secretion in retinal pigment epithelium monolayers. J Biol Chem. (2011) 286:23717–24. 10.1074/jbc.M110.214593 21566137 PMC3129152

[75] Badea T Niculescu F Soane L Fosbrink M Sorana H Rus V. RGC-32 increases p34CDC2 kinase activity and entry of aortic smooth muscle cells into S-phase. J Biol Chem. (2002) 277:502–8. 10.1074/jbc.M109354200 11687586

[76] Badea TC Niculescu FI Soane L Shin ML Rus H. Molecular cloning and characterization of RGC-32, a novel gene induced by complement activation in oligodendrocytes. J Biol Chem. (1998) 273:26977–81. 10.1074/jbc.273.41.26977 9756947

[77] Vlaicu SI Tegla CA Cudrici CD Fosbrink M Nguyen V Azimzadeh P. Epigenetic modifications induced by RGC-32 in colon cancer. Exp Mol Pathol. (2010) 88:67–76. 10.1016/j.yexmp.2009.10.010 19883641 PMC2815209

[78] Fosbrink M Cudrici C Tegla CA Soloviova K Ito T Vlaicu S. Response gene to complement 32 is required for C5b-9 induced cell cycle activation in endothelial cells. Exp Mol Pathol. (2009) 86:87–94. 10.1016/j.yexmp.2008.12.005 19162005 PMC2699899

[79] Fosbrink M Cudrici C Niculescu F Badea TC David S Shamsuddin A. Overexpression of RGC-32 in colon cancer and other tumors. Exp Mol Pathol. (2005) 78:116–22. 10.1016/j.yexmp.2004.11.001 15713436

[80] Wang XY Li SN Zhu HF Hu ZY Zhong Y Gu CS. RGC32 induces epithelial-mesenchymal transition by activating the Smad/Sip1 signaling pathway in CRC. Sci Rep. (2017) 7:46078. 10.1038/srep46078 28470188 PMC5415763

[81] Cho H Lim BJ Kang ES Choi JS Kim JH. Molecular characterization of a new ovarian cancer cell line, YDOV-151, established from mucinous cystadenocarcinoma. Tohoku J Exp Med. (2009) 218:129–39. 10.1620/tjem.218.129 19478469

[82] Donninger H Bonome T Radonovich M Pise-Masison CA Brady J Shih JH. Whole genome expression profiling of advance stage papillary serous ovarian cancer reveals activated pathways. Oncogene. (2004) 23:8065–77. 10.1038/sj.onc.1207959 15361855

[83] Eskandari-Nasab E Hashemi M Rafighdoost F. Promoter Methylation and mRNA expression of response gene to complement 32 in breast carcinoma. J Cancer Epidemiol. (2016) 2016:7680523. 10.1155/2016/7680523 27118972 PMC4828546

[84] Kang Y Siegel PM Shu W Drobnjak M Kakonen SM Cordon-Cardo C. A multigenic program mediating breast cancer metastasis to bone. Cancer cell. (2003) 3:537–49. 10.1016/S1535-6108(03)00132-6 12842083

[85] Hahn A Differentielle Genexpression der Gene APR-1, B56, RGC32 und SIAT-8A bei kutanen T-Zell-Lymphomen. Dissertation, Heidelberg: Ruprecht-Karls-Universität Heidelberg Fakultät für Klinische Medizin Mannheim, (2006).

[86] Schlick S Investigating the role of RGC-32 in cell cycle disruption by EBV EBNA 3C. Dissertation, Sussex: School of Life Sciences, University of Sussex, (2010).

[87] Bredel M Bredel C Juric D Duran GE Yu RX Harsh GR. Tumor necrosis factor-alpha-induced protein 3 as a putative regulator of nuclear factor-kappaB-mediated resistance to O6-alkylating agents in human glioblastomas. J Clin Oncol. (2006) 24:274–87. 10.1200/JCO.2005.02.9405 16365179

[88] Saigusa K Imoto I Tanikawa C Aoyagi M Ohno K Nakamura Y. RGC32, a novel p53-inducible gene, is located on centrosomes during mitosis and results in G2/M arrest. Oncogene. (2007) 26:1110–21. 10.1038/sj.onc.1210148 17146433

[89] Demeure MJ Coan KE Grant CS Komorowski RA Stephan E Sinari S. PTTG1 overexpression in adrenocortical cancer is associated with poor survival and represents a potential therapeutic target. Surgery. (2013) 154:1405–16; discussion 16. 10.1016/j.surg.2013.06.058 24238056 PMC4054940

[90] Zhan F Huang Y Colla S Stewart JP Hanamura I Gupta S. The molecular classification of multiple myeloma. Blood. (2006) 108:2020–8. 10.1182/blood-2005-11-013458 16728703 PMC1895543

[91] Zhu L Qin H Li PY Xu SN Pang HF Zhao HZ. Response gene to complement-32 enhances metastatic phenotype by mediating transforming growth factor beta-induced epithelial-mesenchymal transition in human pancreatic cancer cell line BxPC-3. J Exp Clin Cancer Res. (2012) 31:29. 10.1186/1756-9966-31-29 22458379 PMC3337240

[92] Xu R Shang C Zhao J Han Y Liu J Chen K. Knockdown of response gene to complement 32 (RGC32) induces apoptosis and inhibits cell growth, migration, and invasion in human lung cancer cells. Mol Cell Biochem. (2014) 394:109–18. 10.1007/s11010-014-2086-3 24833469

[93] Schlick SN Wood CD Gunnell A Webb HM Khasnis S Schepers A. Upregulation of the cell-cycle regulator RGC-32 in Epstein-Barr virus-immortalized cells. PLoS ONE. (2011) 6:e28638. 10.1371/journal.pone.0028638 22163048 PMC3232240

[94] An X Jin Y Guo H Foo SY Cully BL Wu J. Response gene to complement 32, a novel hypoxia-regulated angiogenic inhibitor. Circulation. (2009) 120:617–27. 10.1161/CIRCULATIONAHA.108.841502 19652095 PMC2837511

[95] Guo X Jose PA Chen SY. Response gene to complement 32 interacts with Smad3 to promote epithelial-mesenchymal transition of human renal tubular cells. Am J Physiol. (2011) 300:C1415–21. 10.1152/ajpcell.00204.2010 21307346 PMC3118632

[96] Tian J Xu C Yang MH Li ZG. [Overexpression of response gene to complement-32 promotes cytoskeleton reorganization in SW480 cell line]. Nan Fang Yi Ke Da Xue Xue Bao. (2011) 31:1179–82. 21764689

[97] Sun Q Yao X Ning Y Zhang W Zhou G Dong Y. Overexpression of response gene to complement 32 (RGC32) promotes cell invasion and induces epithelial-mesenchymal transition in lung cancer cells via the NF-kappaB signaling pathway. Tumour Biol. (2013) 34:2995–3002. 10.1007/s13277-013-0864-2 23715780

[98] Kim DS Lee JY Lee SM Choi JE Cho S Park JY. Promoter methylation of the RGC32 gene in nonsmall cell lung cancer. Cancer. (2011) 117:590–6. 10.1002/cncr.25451 20862745

